# Oral FXIIa inhibitor KV998086 suppresses FXIIa and single chain FXII mediated kallikrein kinin system activation

**DOI:** 10.3389/fphar.2023.1287487

**Published:** 2023-12-19

**Authors:** Allen C. Clermont, Nivetha Murugesan, Hannah J. Edwards, Daniel K. Lee, Natasha P. Bayliss, Edward J. Duckworth, Stephen J. Pethen, Sally L. Hampton, David Gailani, Edward P. Feener

**Affiliations:** ^1^ KalVista Pharmaceuticals, Cambridge, United Kingdom; ^2^ Hematology/Oncology Division, Vanderbilt University, Nashville, TN, United States

**Keywords:** factor XIIa inhibitor, FXII zymogen, hereditary angioedema, kallikrein-kinin system, HAE, hereditary angioedema

## Abstract

**Background:** The kallikrein kinin system (KKS) is an established pharmacological target for the treatment and prevention of attacks in hereditary angioedema (HAE). Proteolytic activities of FXIIa and single-chain Factor XII (FXII) zymogen contribute to KKS activation and thereby may play roles in both initiating and propagating HAE attacks. In this report, we investigated the effects of potent small molecule FXIIa inhibitors on FXIIa and single chain FXII enzymatic activities, KKS activation, and angioedema in mice.

**Methods:** We examined the effects of 29 structurally distinct FXIIa inhibitors on enzymatic activities of FXIIa and a mutant single chain FXII with R334A, R343A and R353A substitutions (rFXII-T), that does not undergo zymogen conversion to FXIIa, using kinetic fluorogenic substrate assays. We examined the effects of a representative FXIIa inhibitor, KV998086, on KKS activation and both carrageenan- and captopril-induced angioedema in mice.

**Results:** FXIIa inhibitors designed to target its catalytic domain also potently inhibited the enzymatic activity of rFXII-T and the pIC_50_s of these compounds linearly correlated for rFXIIa and rFXII-T (*R* ^2^ = 0.93). KV998086, a potent oral FXIIa inhibitor (IC_50_ = 7.2 nM) inhibited dextran sulfate (DXS)-stimulated generation of plasma kallikrein and FXIIa, and the cleavage of high molecular weight kininogen (HK) in human plasma. KV998086 also inhibited rFXII-T mediated HK cleavage (*p* < 0.005) in plasma from FXII knockout mice supplemented with rFXII-T and stimulated with polyphosphate or DXS. Orally administered KV998086 protected mice from 1) captopril-induced Evans blue leakage in colon and laryngotracheal tissues and 2) blocked carrageenan-induced plasma HK consumption and paw edema.

**Conclusion:** These findings show that small molecule FXIIa inhibitors, designed to target its active site, also inhibit the enzymatic activity of FXII zymogen. Combined inhibition of FXII zymogen and FXIIa may thereby suppress both the initiation and amplification of KKS activation that contribute to hereditary angioedema attacks and other FXII-mediated diseases.

## Introduction

Hereditary angioedema (HAE) is a rare genetic disease that causes spontaneous and unpredictable attacks of swelling affecting skin and submucosal membranes that can be painful, debilitating, and life threatening (Busse and Christiansen, 2020). The most prevalent forms of HAE are associated with genetic mutations in *SERPING1*, which result in the reduced expression of C1 inhibitor or impair its protease inhibitor activity (Drouet et al., 2022). C1 inhibitor is the primary physiological inhibitor of the serine proteases plasma kallikrein (PKa) and factor XIIa (FXIIa) (Davis et al., 2008) and thereby regulates the kallikrein kinin system (KKS). PKa cleaves high molecular weight kininogen (HK) to generate the peptide hormone bradykinin and cleaves zymogen factor XII (FXII) to FXIIa. FXIIa cleaves zymogen plasma prekallikrein (PK) to PKa and thereby activates and amplifies the KKS. Insufficiently controlled PKa and FXIIa activities due to C1 inhibitor deficiency can result in high concentrations of bradykinin that can cause vascular hyperpermeability, inflammation, and edema (Schmaier, 2016). PKa inhibitors have been shown to be effective for the treatment and prevention of HAE attacks (Marco et al., 2010; Banerji et al., 2018; Zuraw et al., 2021; Aygören-Pürsün et al., 2023). Since FXIIa plays a critical role in KKS activation, inhibitors of FXIIa may provide a therapeutic approach to prevent uncontrolled activation of the KKS that leads to HAE attacks. A recent phase 3 study in HAE demonstrated that monthly administration of garadacimab, a monoclonal antibody that inhibits FXIIa, resulted in 87% attack reduction with 62% of patients being attack free over the six-month study (Craig et al., 2023). These effects of garadacimab were comparable with the clinical efficacy of the monoclonal antibody PKa inhibitor lanadelumab, which provided 87% HAE attack reduction with 44% of patients attack free in a 26-week phase 3 study (Banerji et al., 2018). The comparable clinical efficacies of FXIIa and PKa inhibition on the prevention of HAE attacks by garadacimab and lanadelumab, respectively, suggests that activated FXII contributes to most or all KKS actions that mediate HAE attacks.

It has been proposed that the intrinsic enzymatic activity of FXII plays a role in initiation of contact system activation (Ivanov et al., 2017) and thereby FXII zymogen may contribute to triggering HAE attacks. Single-chain FXII in solution predominantly exists in a ‘closed’ conformation and binding to an anionic surface or macromolecule such as polyphosphate stabilizes an ‘open’ conformation that facilitates cleavage by prekallikrein (Shamanaev et al., 2023). Although the intrinsic enzymatic activity of FXII is several orders of magnitude lower than that of FXIIa (Ivanov et al., 2017) its activity is sufficient to mediate activation of prekallikrein.

Orally administered FXIIa inhibitors may provide a non-invasive approach to control the KKS in HAE and thereby prevent attacks. In contrast to its proximate proteases in the KKS and intrinsic coagulation pathway, namely, PKa and coagulation factor XIa (FXIa), for which multiple oral small molecules have been discovered and advanced to clinical studies (Busse and Kaplan, 2022; Greco et al., 2023), few small molecule inhibitors of FXIIa have been described. Moreover, little is known about the pharmacological inhibition of FXII zymogen and how it compares with FXIIa. Using structure-based drug discovery we have identified a portfolio of potent small molecule FXIIa inhibitors. We compared the effects of these FXIIa inhibitors on the enzymatic activities of FXIIa and FXII-T, a FXII mutant that has alanine substitutions for arginine at the activation cleavage sites (Arg334, Arg344 and Arg353) (Ivanov et al., 2017) that prevent zymogen conversion to FXIIa. The pharmacology of a representative orally available FXIIa inhibitor, KV998086, was examined on KKS activation in plasma and on angioedema in mice.

### Methods

#### Identification of small molecule FXIIa inhibitors

Using structure-based design a portfolio of novel potent FXIIa inhibitors targeting its catalytic site were identified. Iterative design-make-test-analyze cycles were used to optimize for drug metabolism and pharmacokinetics (DMPK) SAR while maintaining and improving FXIIa potency and selectivity. KV998086, an orally available competitive FXIIa inhibitor, was identified from a lead compound series established through a fragment growth approach. KV998086 is Lipinski compliant, with a MW < 500 Da, measured logD at pH 7.4 of 2.58, two hydrogen bond donors (HBD) and eight hydrogen bond acceptors (HBA). Rigidification of the scaffold, reducing rotatable bonds to 5, also benefited oral exposure and contributed to the high ligand efficiency (LE) of KV998086 (LE = 0.33).

#### Generation of recombinant single chain FXII

Human recombinant rFXII-T is a mutant form of FXII with R334A, R343A and R353A substitutions that does not undergo auto-catalysis to FXIIa in the presence of polyphosphates (PolyP) and is resistant to cleavage by FXIIa and PKa (Ivanov et al., 2017). Recombinant rFXII-T and wild-type (rFXII-WT) were generated in HEK293-6E cells (Proteos, Michigan, United States) and isolated from the culture media. Proteins were purified by ion exchange chromatography (SP Sepharose FF) and eluted fractions containing rFXII-T were pooled, concentrated using spin columns, and sterile filtered through a 0.2-micron PES membrane. The final buffer contents included 4 mM sodium acetate and 150 mM NaCl, pH 5.2. Enzyme activity assays with rFXII-T were performed as described below.

#### Protease IC_50_ determination

Effects of a panel of 29 structurally diverse, small molecule FXIIa inhibitors, ranging in molecular weight from 401-685 Da, calculated logarithm of the octanol−water partition coefficient (cLogP) 1.2–5.6, HBA 7-11, HBD 2–4 and topological polar surface area (TPSA) 75-131Å^2^, with FXIIa IC_50_s ranging from 0.31 nM to 308 nM, on the catalytic activities of human recombinant rFXIIa (1nM, Molecular Innovations) and human recombinant rFXII-T (200 nM) were determined using kinetic substrate cleavage assays using fluorogenic substrate H-D-Pro-Phe-Arg-AFC (Peptide Protein Research Ltd., United Kingdom) performed at 37°C for 5 min and 60 min, respectively, for rFXIIa and rFXII-T. Protease activities were measured by monitoring the accumulation of liberated fluorescence from the substrate using a SPARK fluorimeter (Tecan, United Kingdom). The rate of fluorescence increase per minute was expressed as percentage of control activity (no inhibitor).

The Km for the cleavage of substrate was determined by standard transformation of the Michaelis-Menten equation using GraphPad Prism (GraphPad Software, Inc., La Jolla, United States). The Km of H-D-Pro-Phe-Arg-AFC for FXIIa was 288 nM ± 2.3 nM and for FXII-T was 223 ± 48.6 nM. The compound inhibitor IC_50_ assays were performed at substrate K_m_ concentration, as a 10-point dose response curve, in duplicate, using ½ logarithmic concentrations up to 4 µM. Compound was pre-incubated with each enzyme 5 min before substrate addition. Activities were calculated at the concentration of inhibitor that gave 50% inhibition (IC_50_) of the uninhibited control enzyme activity (100%) using a four-parameter logistic equation.

Inhibitory activity of KV998086 was determined on FXIIa alpha generated from FXII that was purified from human plasma (pFXIIa) and from mouse plasma (HFXIIa 1212a, Enzyme Research Laboratories, United Kingdom; MFXIIa, Molecular Innovations) at 25°C, at an enzyme concentration of 1 nM using the IC_50_ method described. Effects of KV998086 on amidolytic activity in stimulated human pooled plasma (control plasma, Affinity Biologicals, Canada) were measured using the fluorescent substrate H-D-Pro-Phe-Arg-AFC (Peptide Protein Research Ltd., United Kingdom), which is cleaved by both FXIIa and PKa. The KKS in control plasma was stimulated by the addition of dextran sulfate 500 kDa (DXS; Sigma-Aldrich, United States) 6.25 μg/mL at 4°C (Duckworth et al., 2022) or long chain polyphosphates with approximate polymer lengths ranging from ∼200-1,300 phosphate units (PolyP, p700, Kerafast, United States) 1 mg/mL at 37°C. PKa activity in the plasma was estimated based on the maximum rate of fluorescence increase. To determine the effect of FXIIa inhibition on downstream PKa activity, KV998086 (at 8 concentrations, in ½ log up to 40 µM) was preincubated for 30 min in control plasma prior to KKS stimulation. The normalized maximum rate of fluorescence increase data were fitted to a 4-parameter logistic dose response curve to estimate the IC_50_.

#### Capillary-based immunoassay of KKS components

Dose-response experiments were performed on control plasma pretreated with KV998086 (concentrations ranging from 30 to 1000 nM, total n = 6 experiments) for 15 min followed by stimulation with 6.25 μg/mL DXS at 4°C for 17 min to induce KKS activation and PKa-mediated HK cleavage. Reactions were stopped by addition of Laemmli buffer containing β-mercaptoethanol and heating samples at 95°C for 10 min. Components of the KKS in plasma samples (diluted 1:20) were separated by capillary electrophoresis using 12-230 kDa separation modules in the Protein Simple WES™ immunoblotting system (ProteinSimple, CA, United States). Proteins were detected by immunoassay using primary antibodies for HK and cHK (KNG17A12, Innovative Research, United States), PK and PKa (Ab44392, Abcam, United States), FXII and FXIIa (GAFXII-IG, Affinity Biologicals, Canada), and horseradish peroxidase-conjugated secondary antibody. Proteins were quantified from peak areas corresponding to chemiluminescence intensities using Compass for Simple Western software (Version 4.0.0, ProteinSimple) and visualized as virtual blots generated from capillary electropherograms as previously described (Duckworth et al., 2022).

#### Recombinant FXII-T mediated KKS activation

rFXII-T (200 nM) was incubated with 200 nM plasma prekallikrein and stimulated with long-chain polyphosphates (70 μM, PolyP, p700, Kerafast) at 37°C. Concentration response experiments with KV998086 were conducted at 37°C for 180 min. Reactions were stopped by rapidly heating samples to 95°C for 10 min in Laemmli buffer containing β-mercaptoethanol. For HK cleavage studies in mouse plasma, rFXII-T (200 nM) was incubated with FXII^−/−^ plasma and PolyP (10 μg/mL) or dextran sulfate, DXS (40 kDa, 2.5 μg/mL) for 2 h at 37°C (Pawaskar et al., 2021). Concentration response experiments were conducted at 0.5, 5 and 15 μM KV998086. Reactions were stopped by rapidly heating samples to 95°C for 10 min in Laemmli buffer containing β-mercaptoethanol.

#### Animal description

All murine study protocols were reviewed and approved by the Institutional Animal Care and Use Committee (IACUC) of Mispro Contract Vivarium (Cambridge, MA) accredited by the Association for Assessment and Accreditation of Laboratory Animal Care (AAALAC). Studies were conducted by KalVista Pharmaceuticals in accordance with the United States National Research Council’s Guide for the Care and Use of Laboratory Animals, ARRIVE 2.0 guidelines and the recommendations of Frontiers in Medicine. Male (25.7 ± 0.4 g) and female (20.1 ± 0.4 g) C57BL/6J mice at 2–3 months of age were obtained from Jackson Laboratories (Bar Harbor, ME, United States). Mice were acclimated to the vivarium for 1 week prior to entry into studies. FXII^−/−^ mice were obtained from Innovative Research (Novi, MI, United States). FXII^−/−^ mice were backcrossed with wild-type (WT) C57BL/6J for nine generations to achieve 99.4% C57BL/6J genetic background. Genotyping of FXII^−/−^ mice was confirmed using real-time PCR by Transnetyx, Inc. (Cordova, United States). Male (25.2 ± 1.5 g) and female (20.2 ± 0.3 g) homozygous FXII^−/−^ mice at 2–3 months of age were utilized as a FXII deficiency control. All mice were housed in standard caging (4/cage) with water/food *ad libitum* and a 12-h light/dark cycle. *In vivo* studies were powered for sample size using SigmaPlot v12.5 statistical software. The number of mice per group were determined by a one-way ANOVA comparison based on a minimum detectable difference of the means with a power of 0.8 and alpha = 0.05. Mice were weighed and randomized according to body weight at study onset. The total number of mice per group were compiled from multiple independent experiments. Mice were excluded from a study if any adverse events were observed due to drug delivery or surgical interventions. All assay samples were collected and masked for study analyses.

#### *In vivo* administration of KV998086

KV998086 was administered at 2.4, 7.2 or 18 mg/kg/day or vehicle alone (10% DMSO (Sigma, United States), 10% Cremophor EL (Sigma, United States) and 80% water) via mini-osmotic pumps (model 1003D, Alzet, United States). Prior to surgical procedure for pump implantation, mice received a subcutaneous injection of Meloxicam (Metacam Injectable, Boehringer-Ingelheim, United States) at 5 mg/kg (1:10 dilution in saline at 0.5 mg/mL) for analgesia. Under 2.5% isoflurane inhalation the flank area was shaved and prepared with Betadine. Pumps were implanted into a subcutaneous pocket in the flank 48 h prior to the start of subsequent interventions. Surgical sites were monitored daily for infection or pump exposure. For oral studies, mice received a gavage of KV998086 (in 10% DMSO, 10% Cremophor EL and 80% water) via 20G feeding needle at 5, 25 or 45 mg/kg at a stock concentration of 2 or 6 mg/mL or vehicle alone.

#### Carrageenan induced paw angioedema and systemic KKS activation

The carrageenan-induced paw edema model was utilized to examine the systemic activation of KKS and its role in tissue swelling. Lambda-carrageenan (CG, Sigma, United States) was prepared in water, warmed at 37°C for 2 h, agitated until thoroughly dissolved, and kept at 21°C for up to 48 h prior to use. Under isoflurane anesthesia, male mice received a 25 µL injection (29G, 0.3 mL syringe) of CG into the subplantar region of the hind paw and vehicle alone injected into the contralateral paw. Effects of KV998086 on paw edema and systemic KKS activation in the plasma were examined in mice using two concentrations of CG. A 2% CG solution was used for measurements of paw edema at 24 h post injection. A 0.5% CG solution was used for measurements of KKS activation in plasma and paw edema at 2 h post injection. Paw thickness was measured using a digital caliper (Mitutoyo, Kanagawa, Japan) to estimate paw edema by measuring central hind paw width and thickness. Upon termination of the study, mice were anesthetized with 5% isoflurane. A laparotomy was performed to expose the inferior vena cava and blood samples were collected via a 26G 1 cc syringe coated with 3.2% sodium citrate. After blood collection, mice were euthanized by thoracotomy as specified by the American Veterinary Medical Association Guidelines for the Euthanasia of Animals. Plasma was obtained and KKS analyses were performed as described above.

#### Captopril-induced vascular permeability in mice

Female mice (WT and FXII^−/−^) were randomized into pretreatment groups with vehicle or KV998086 administered via Alzet pump or oral gavage, as described above. While under 2.5% isoflurane anesthesia, the right jugular vein was cannulated with a sterile polyurethane catheter (Micro-Renathane MRE025 0.635 mm OD, Braintree Scientific, Braintree MA, United States) filled with sterile saline. Through the catheter, captopril (Sigma-Aldrich, United States) was infused at 2.5 mg/kg followed by EB (Sigma-Aldrich, United States) dye at 30 mg/kg. After 30 min, mice were euthanized by CO_2_ asphyxiation and perfused through the left ventricle with 10 mL PBS by a 25G butterfly needle to remove vascular EB. Colon and laryngotracheal (larynx and adherent trachea) tissues were collected and frozen. Tissue weights were measured after drying the colon and larynx + trachea in a vacufuge (Eppendorf, United States) for 4 h at 60°C and incubated in formamide at 72°C overnight. Samples were then centrifuged and the absorbance in supernatant was measured at 620 nm and 740 nm (Spark, TECAN, Austria). EB concentration was interpolated in the samples using a standard curve and normalized by dry weight of the tissue (ng/mg tissue weight).

#### Statistics

Data were analyzed using GraphPad Prism v8.4.3 and presented as mean ± standard error of the mean (SEM). For *in vitro* studies, group statistics were determined by one-way ANOVA compared to control. Significant differences (*p* < 0.05) were determined using uncorrected Fisher’s least significant differences (LSD) test. For *in vivo* studies, group statistics were determined by one-way ANOVA for multiple comparisons. Brown-Forsythe and Bartletts Tests were used to determine equality of variances for all groups. Significant differences (*p* < 0.05) were determined using Dunnett’s multiple comparison test.

## Results

### Inhibition of FXIIa and FXII single chain enzyme activities

**FIGURE 1:**
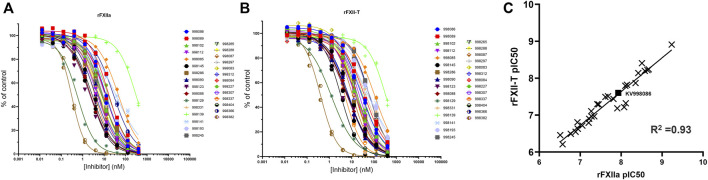
Effects of FXIIa inhibitors on rFXIIa and rFXII-T amidolytic activities. Inhibition curves for 29 small molecule FXIIa inhibitors, indicated by compound identifying numbers, for **(A)** human rFXIIa (1 nM) and **(B)** single chain rFXII-T (200 nM). Amidolytic activities were measured using a fluorogenic substrate. Data is plotted as percent of control reative to rFXIIa or rFXII-T in the absence of inhibitor. **(C)** Correlation between the pIC50 for rFXIIa versus rFXII-T for all compounds tested.

**FIGURE 2:**
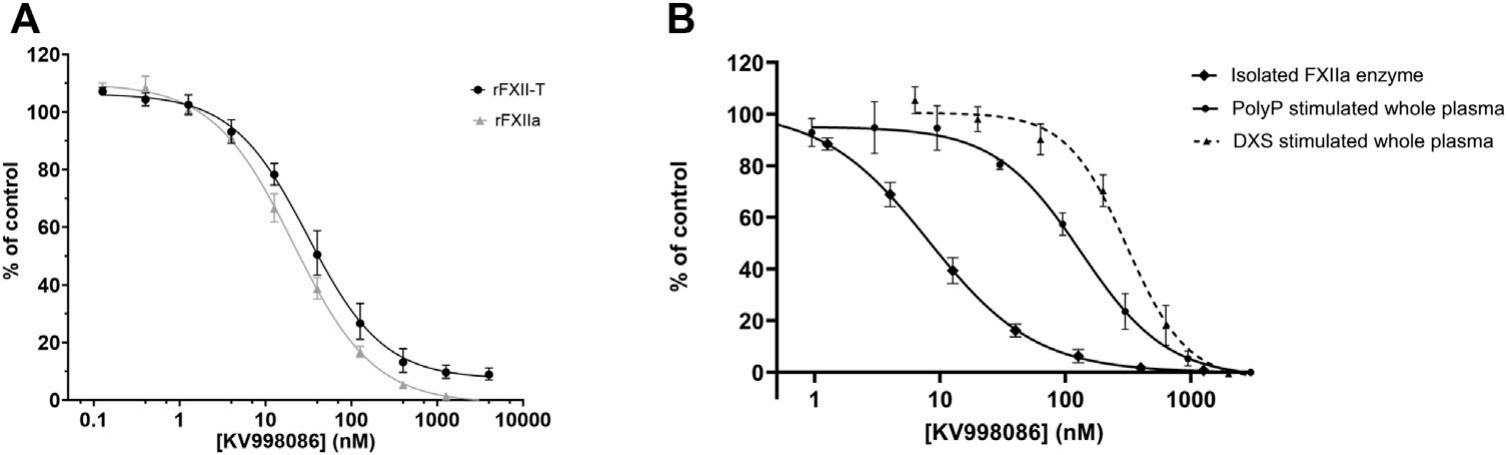
Effects of KV998086 on isolated rFXIIa and rFXII-T and activated plasma **(A)** Effects of KV998086 on enzyme activities of rFXIIa (1 nM) and rFXII-T (200 nM). **(B)** Effects of KV998086 on amidolytic activity of FXIIa isolated from human plasma and activated human plasma. Plasma derived FXIIa was pretreated for 5 min with the indicated concentrations of KV998086. Plasma was pre-treated with KV998086 for 30 min prior to stimulation by dextran sulfate (DXS) or long-chain polyphosphate (PolyP). Amidolytic activity was measured using a fluorogenic substrate. Data are plotted as percent of control related to FXIIa or activated plasma in the absence of KV998086.

A panel of novel and structurally diverse small molecule FXIIa inhibitors were screened against recombinant human rFXIIa and rFXII-T to measure their potency in inhibiting enzyme activities measured by a fluorogenic substrate assay. As shown in Figures 1A,B, all 29 compounds inhibited enzyme activity for both rFXIIa and rFXII-T in a dose dependent manner. Inclusion of 70 μM PolyP did not increase enzyme activity of isolated rFXIIa or rFXII-T measured using the flurogenic substrate, hence assays were performed without PolyP. Comparison of the pIC50s of compounds for rFXIIa and rFXII-T demonstrated a linear correlation (*R* ^2^ = 0.93), with comparable IC_50_s (Figure 1C). KV998086, an oral non-peptidic FXIIa inhibitor, was selected for further studies. Its IC_50_s for rFXIIa and rFXII-T are 21 ± 2.4 nM and 31 ± 4.0 nM respectively (Figure 2A). The IC_50_ of KV998086 for FXIIa generated from FXII isolated from human plasma (pFXIIa) is 7.2 ± 2.2 nM (n = 19) (Figure 2B) and for mouse pFXIIa is 9.4 ± 2.2 nM (n = 5) (Supplementary Figure S1). KV998086 inhibited DXS- and PolyP-stimulated PKa activity in human plasma with mean IC_50_s of 305 ± 45 nM (SD, n = 9) and 133 ± 14 nM (SD, n = 4), respectively (Figure 2B). Dextran sulfate is a much more powerful inducer of contact activation than is polyphosphate, which results in greater FXIIa activity generated in the DXS assay. In addition, plasma was diluted 3:5 in the PolyP assay and 9:10 in the DXS assay. These factors may explain the different observed IC_50_s values for KV998086 in these plasma-based assays.

KV998086 selectivity for FXIIa is > 1000-fold compared with the IC_50_ of a panel of 15 human serine proteases, including FXa and FXIa (IC_50_ > 40 µM), thrombin (IC_50_ 16 µM) and tissue kallikrein 1 (IC_50_ 7.9 μM) (Supplementary Table S1). The measured human plasma protein free fraction for KV998086 is 0.118, which likely contributes to the right shift in IC_50_ in the plasma assays compared to measurements using isolated FXIIa (Supplementary Table S2).

### KV998086 inhibits DXS-stimulated KKS activation in plasma

**FIGURE 3:**
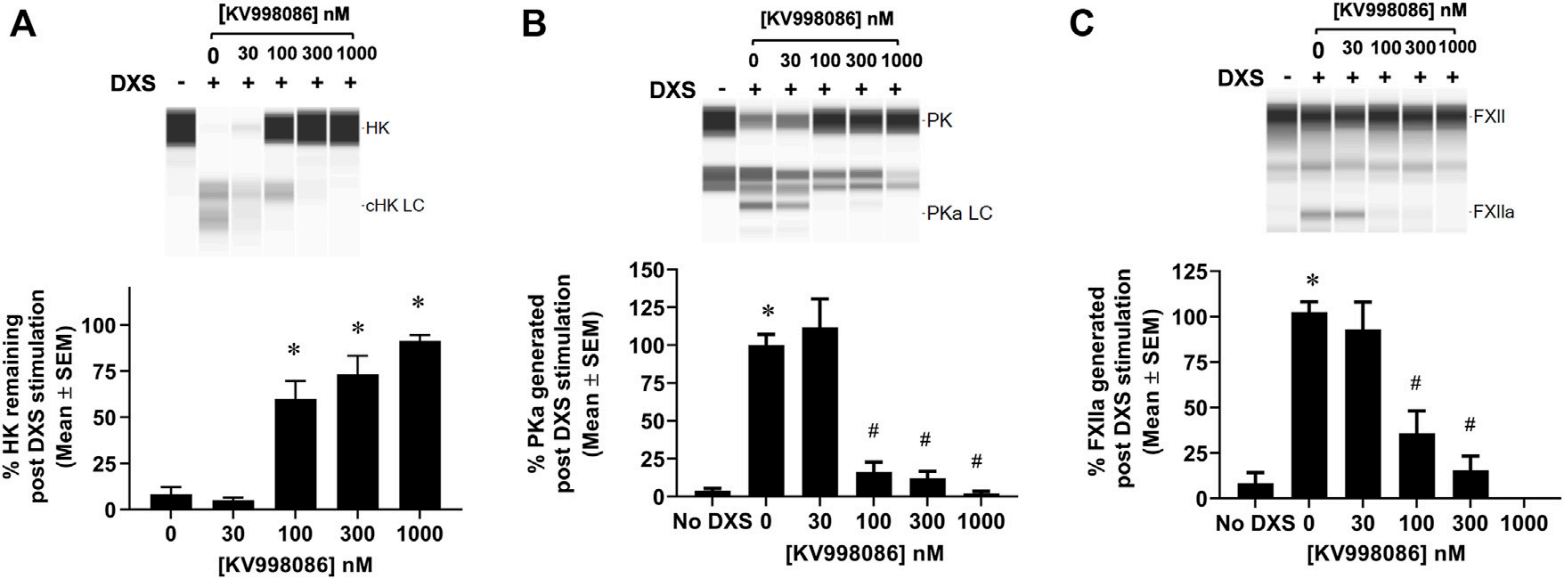
Effects of KV998086 on the kallikrein kinin system activation in DXS-stimulated human plasma. HK, PKa and FXIIa in plasma, in the absence or presence of KV998086, were measured 17 min after the addition of DXS (6.25 μg/mL). A representative image generated from capillary electropherograms are shown. Bar graphs show the percent of **(A)** HK relative to samples without DXS stimulation and both **(B)** PKa and **(C)** FXIIa relative to DXS-stimulated samples without KV998086. Results are expressed as % mean ± SEM from 6 independent experiments. *p < 0.05 as compared to HK remaining in samples with DXS stimulation but no KV998086, or FXIIa and PKa present in samples without DXS stimulation. #*p* < 0.05 as compared to FXIIa and PKa generated in samples with DXS stimulation without KV998086.

The effects of KV998086 on DXS-stimulated HK cleavage and the generation of PKa and FXIIa were quantified in human plasma. In the absence of KV998086, DXS-stimulation decreased HK by 91% (8.3% ± 3.9% HK remaining, SEM, n = 6), relative to unstimulated plasma. KV998086 inhibited DXS-stimulated HK cleavage in a concentration-dependent manner (IC_50_ of 82 nM) with significant protection at ≥100 nM (*p* ≤ 0.0001, Figure 3A). DXS-stimulated generation of PKa was inhibited by KV998086 with a mean IC_50_ of 60 nM and with inhibition of 83.5% at 100 nM, relative to DXS-stimulated plasma without inhibitor (*p* ≤ 0.0001, Figure 3B). The lower IC_50_ of KV998086 for DXS-stimulated PKa generation compared with its IC_50_ for DXS-stimulated PKa enzymatic activity (Figure 2B) is attributed to different methodologies, which include differences in assay temperatures, substrates, and rate vs. endpoint measurements. KV998086 inhibited DXS-stimulated generation of FXIIa with an IC_50_ of 81 nM and provided 84% inhibition at 300 nM (*p* ≤ 0.0001, Figure 3C).

### Effect of KV998086 on PolyP stimulated PKa generation by FXII-T

**FIGURE 4:**
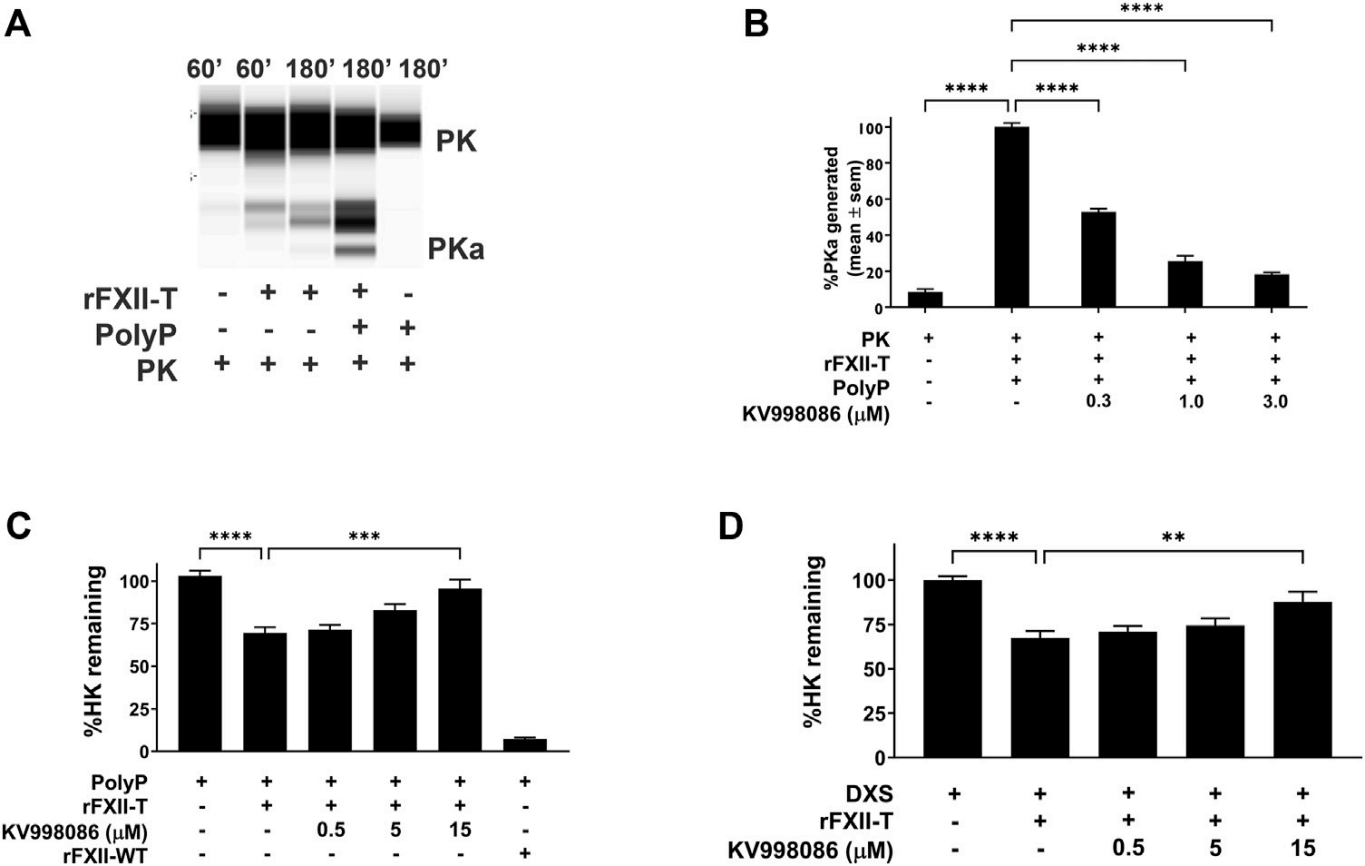
Effects of KV998086 on FXII-T mediated kallikrein kinin system activation. **(A)** Effects of FXII-T in the absence or presence of PolyP on the cleavage of PK to PKa at 37°C **(B)** Effect of KV998086 on PolyP stimulated PKa generation from PK by FXII-T activity. Dose response effects of KV998086 on FXII-T stimulated HK cleavage in FXII^−/−^ mouse plasma with two activators: long chain PolyP **(C)** and DXS **(D)** are shown. **p = 0.0013, ****p < 0.0001 as compared to HK remaining in samples with PolyP or DXS stimulation without KV998086.

In the absence of PolyP, rFXII-T generates a small amount of PKa from plasma prekallikrein (PK) when co-incubated at 37 °C for 60 min. In the presence of PolyP, rFXII-T mediated cleavage of PK leads to increased PKa generation by 180 min of incubation at 37°C, compared to control without PolyP (Figures 4A, B *p*< 0.0001). Incubation of PK with PolyP alone did not generate PKa in this assay. KV998086 inhibits rFXII-T mediated PKa generation in a concentration-dependent manner (*p* < 0.0001) (Figure 4B).

### FXII-T mediated KKS activation when spiked in FXII^−/−^ mouse plasma

Addition of PolyP alone did not stimulate HK cleavage in FXII^−/−^ mouse plasma (Figure 4C). Addition of PolyP to FXII^−/−^ plasma containing rFXII-WT (wild-type zymogen) or rFXII-T stimulated HK cleavage by 92.8% (*p* < 0.0001) and 32% (*p* < 0.01), respectively (Figure 4C). KV998086 inhibited PolyP stimulated HK cleavage in FXII^−/−^ plasma spiked with rFXII-T in a concentration dependent manner (*p* = 0.0001, 15 μM, Figure 4C). KV998086 also inhibited DXS stimulated HK cleavage in FXII^−/−^ plasma spiked with rFXII-T (*p* = 0.0013, 15 μM, Figure 4D).

### Pharmacokinetic profile and bioavailability of KV998086

The pharmacokinetic profiles for a single oral gavage of KV998086 are shown for four nonclinical species in Supplementary Figure S2. Time to peak concentration (t_max_) was 120, 90, 300 and 240 min for mouse (A), dog (B), NHP (C) and rat (D), respectively. Bioavailability (F%) was calculated as 78, 75% and 92% in rat, dog and NHP, respectively. The oral half-life was 3.4, 6.7 and 13.6 h in rat, NHP and dog, respectively (Supplementary Table S2).

### KV998086 protects against carrageenan-induced paw edema and systemic KKS activation

Carrageenan (CG) is a mixture of natural linear sulfated polysaccharides from seaweed that have been shown to induce inflammation and paw edema mediated, in part, by PKa (Kenniston et al., 2014). Injection of a 2% solution of CG into the paw caused a 74.8% increase in the paw thickness measured 24 h post injection compared to paws injected with water. Pretreatment with KV998086 (7.2 mg/kg/day via osmotic pumps) reduced CG stimulated paw thickening by 47.8% (*p* < 0.01) (Supplementary Figure S3). In mice with oral gavage of vehicle delivered at 2 h prior, CG injection at 0.5% caused a 52.9% increase in paw thickness measured at 2 h post injection compared to paws injected with water. Oral gavage of KV998086 (45 mg/kg, delivered 2 h prior to CG) resulted in a 37.0% (*p* < 0.01) decrease in paw thickening at 2 h post CG injection (Supplementary Figure S3). KV998086 is as effective as complete FXII deficiency (FXII^−/−^ mice) in protecting against CG-stimulated paw thickening.

**FIGURE 5:**
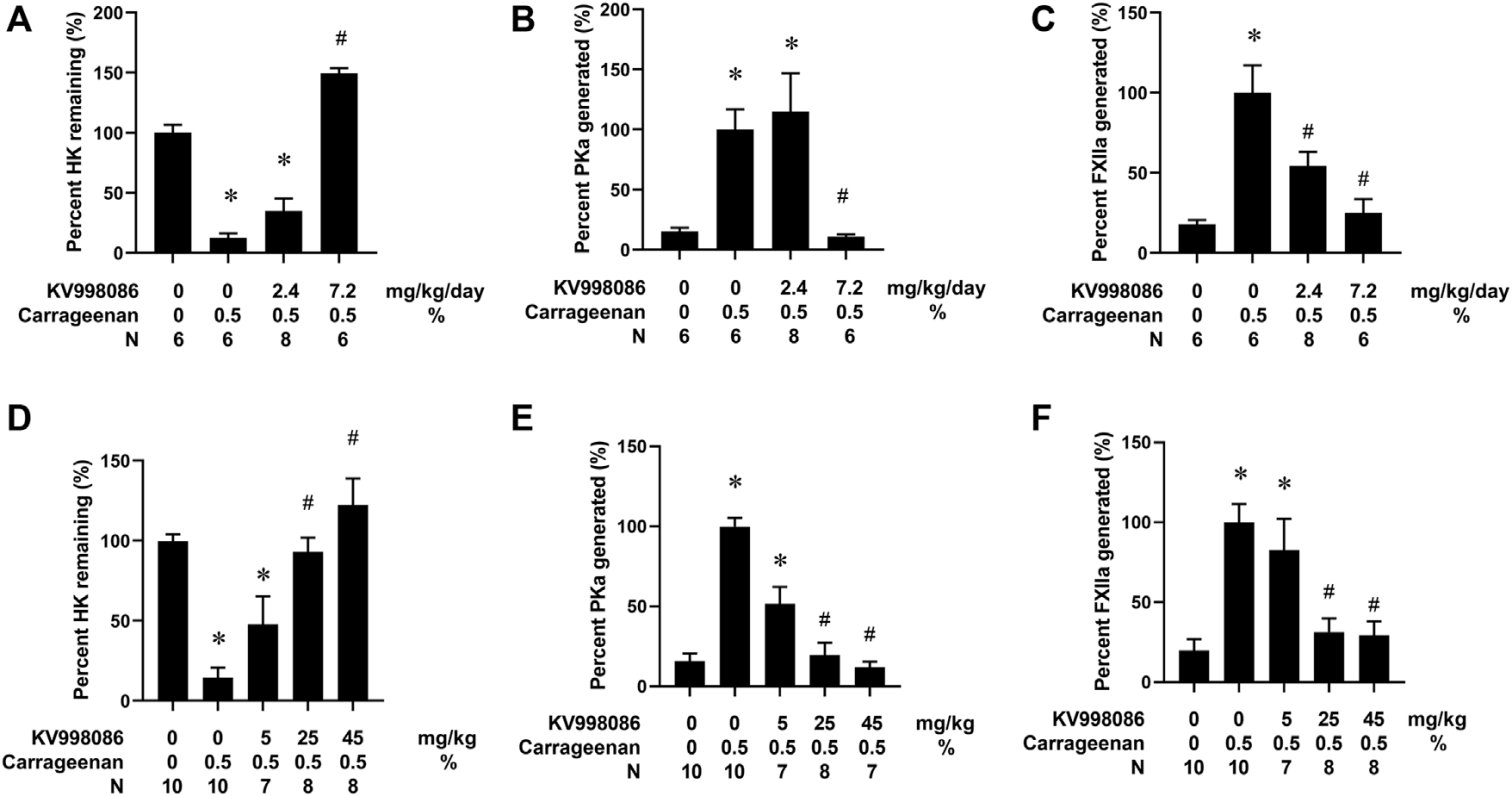
Effects of KV998086 on carrageenan (CG)-stimulated kallikrein kinin system activation. WT mice were subcutaneously implanted with osmotic pumps to deliver KV998086 at rates of 0, 2.4 and 7.2 mg/kg/d at 48 h prior to hind paw injections. 25µL of 0.5% CG or water were injected in contralateral hind paws and plasma collected at 1.5 h post injections. Protein levels of HK, PKa, and FXIIa in plasma were detected by immunoassay. Data were obtained from four independent experiments with total number of animals per group indicated. **(A)** The percentage of HK relative to samples from mice that did not receive CG. **(B)** PKa **(C)** FXIIa show percentages relative to CG-stimulated samples without inhibitor. Results are expressed as % mean ± SEM. *p < 0.05 as compared to vehicle alone, #*p* < 0.05 as compared to CG alone. Effect of oral KV998086 on CG mediated kallikrein kinin system activation in plasma. WT mice received oral gavage of KV998086 at doses of 5, 25 and 45 mg/kg administered 2 h prior to hind paw injections of 25 µL of 0.5% CG. Plasma was collected at 1.5 h post CG injection. Protein levels of HK, PKa and FXIIa in the plasma were detected by immunoassay. Data were obtained from 5 independent experiments. **(D)** The percentage of HK relative to samples from mice that did not receive CG. **(E)** PKa and **(F)** FXIIa show percentages relative to CG-stimulated samples without inhibitor. Results are expressed as % mean ± SEM. *p < 0.05 as compared to vehicle alone, #*p* < 0.05 as compared to CG alone.

CG injection in the paw resulted in an 87.4% reduction (*p* < 0.05) in circulating HK in plasma compared with mice receiving a paw injection with water (Figure 5A). CG injection in the paw also increased both PKa and FXIIa by 6.6- and 5.6- fold, (*p* < 0.05), respectively, in the plasma compared with water injected control mice (Figures 5B, C). Osmotic pump delivery of KV998086 protected mice from CG-stimulated HK cleavage in a dose dependent manner with complete inhibition observed at 7.2 mg/kg/day dosing (*p* < 0.001) and corresponding reductions in PKa (*p* < 0.01) and FXIIa (*p* < 0.001). Administration of KV998086 with osmotic pumps at 7.2 mg/kg/day achieved a steady state plasma concentration of 165 ± 15.5 ng/mL (Supplementary Figure S4) that inhibited CG-induced KKS activation. In mice receiving an oral gavage of vehicle, CG injection in the paw resulted in an 85.5% reduction in circulating HK and an increase in PKa and FXIIa by 6.2 and 5.1-fold, respectively, in plasma compared with mice receiving a paw injection with water (*p* < 0.05) (Figures 5D, E, F). A dose response effect of orally administered KV998086 on the inhibition of systemic KKS activation was observed in mice subjected to paw injection with CG. Oral doses of 25 mg/kg KV998086 and higher provided protection from CG-induced HK cleavage and PKa and FXIIa generation measured in plasma (*p* < 0.05 compared to CG control).

### KV998086 inhibits captopril-induced Evans blue leakage in mice

**FIGURE 6:**
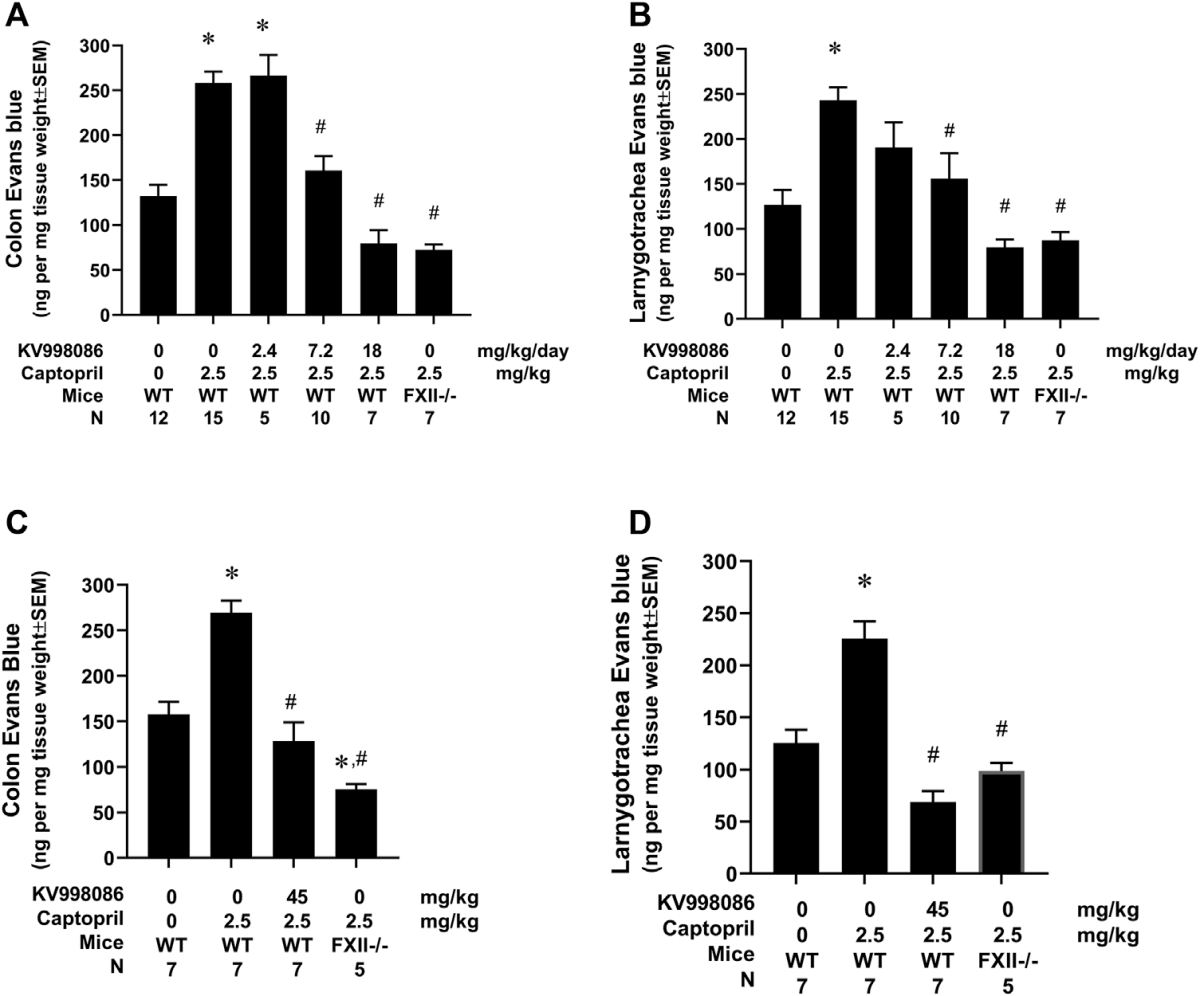
Effect of KV998086 on captopril induced extravasation of Evan’s blue dye. KV998086 was delivered via osmotic pumps at 2.4, 7.2 or 18 mg/kg/day for 48 h prior to IV infusion of captopril (2.5 mg/kg) or vehicle in WT and FXII^−/−^ mice. Evan’s blue (EB) content was quantified from colon **(A)** and larnygotracheal tissues **(B)** obtained at 0.5 h after IV infusion. Data were obtained from 8 independent experiments and the number of animals per group are shown. KV998086 was delivered via oral gavage at 45 mg/kg at 15 h prior to IV infusion of captopril (2.5 mg/kg) or vehicle in WT mice. EB content was quantified from colon **(C)** and larnygotracheal tissues **(D)** obtained at 0.5 h after IV infusion. Data were obtained from five experiments and the number of animals per group are shown. Data are expressed as ng EB per mg tissue and expressed as mean ± SEM. (*p < 0.05 as compared to vehicle control, #*p* < 0.05 as compared to captopril control).

The effects of KV998086 administered via subcutaneously implanted osmotic pumps on captopril-induced vascular permeability to EB were measured in mice. Infusion of captopril induced a 1.96-fold increase in EB leakage in the colon compared to control mice infused with saline (258.2 ± 12.9 ng/mg tissue weight versus 131.9 ± 12.9 ng/mg, *p* < 0.001). This effect of captopril on EB leakage was reduced by 77.3% in mice pretreated with 7.2 mg/kg/day of KV998086 and by 142% in mice receiving 18 mg/kg/day KV998086 (*p* < 0.001, Figure 6A). In laryngotracheal tissue, captopril caused a 1.91-fold increase in EB leakage (242.9 ± 14.5 ng/mg tissue weight) relative to control animals receiving saline (126.7 ± 16.5 ng/mg, *p* < 0.001). Captopril induced vascular leakage was reduced by 75.0% at 7.2 mg/kg/day (*p* < 0.01) and by 140% at 18 mg/kg/day KV998086 (*p* < 0.001, Figure 6B). FXII^−/−^ fully protected both colon (147%) and laryngotracheal (134%) (*p* < 0.001) tissue from captopril-induced leakage.

The effects of orally administered KV998086 on captopril-induced vascular permeability were measured by EB in mice. Pretreatment of mice with 2 doses of vehicle delivered over a 15-h period followed by infusion of captopril induced a 1.7-fold (269.2 ± 13.5 ng/mg tissue weight versus 157.7 ± 13.8 ng/mg) and 1.8-fold (225.6 ± 12.6 versus 125.5 ± 12.6 ng/mg) increase in EB leakage in the colon and laryngotracheal tissue, respectively, compared to control mice infused with saline. Pretreatment of mice with 2 doses of orally administered KV998086 (45 mg/kg) reduced captopril stimulated vascular leakage in the colon by 126.3% (128.4 ± 20.5 ng/mg tissue weight) and by 156% (68.9 ± 10.5 ng/mg) in the laryngotracheal tissue compared to captopril-stimulated control mice receiving oral vehicle alone (Figures 6C, D). Mice receiving oral KV998086 and FXII^−/−^ mice were similarly protected from captopril-stimulated EB leakage.

## Discussion

This report demonstrates that small molecule inhibitors of FXIIa designed to target its catalytic site also inhibit the enzymatic activity of single-chain FXII-T. Moreover, we show a representative oral FXII/FXIIa inhibitor, KV998086, inhibits the generation of both PKa and FXIIa and blocks HK cleavage in DXS-stimulated human plasma and in mice subjected to CG-induced inflammation. Orally administered KV998086 was as effective as FXII deficiency (FXII^−/−^) in preventing both CG- and captopril-induced edema in mice. These results provide the first demonstration of an oral FXII/FXIIa inhibitor that provides protection against KKS activation and edema.

Studies using FXIIa inhibitor antibodies, proteins, and cyclic peptides have demonstrated the important role of FXIIa in a variety of diseases using animal models (May et al., 2016; Cao et al., 2018; Wilbs et al., 2020). However, protein and peptidic inhibitors of FXIIa require parenteral administration that may limit their clinical utility for the treatment of FXIIa-mediated diseases. We sought to identify orally available FXIIa inhibitors that potently and selectively target the enzyme active site. To enable structure based-drug design in our discovery program, we used a soaking approach to generate protein crystals of β-FXIIa in complex with our compounds in its catalytic site, following a protocol recently described (Dementiev et al., 2018; Pathak et al., 2019). The reported structures of β-FXIIa in complex with ligands, benzamidine and D-Phe-Pro-Arg chloromethyl ketone, revealed a deep and well-defined specificity pocket (S1) and a closed S2 pocket resulting in large, open, and solvent exposed S4 and prime regions. These features created a challenge for medicinal chemistry to obtain sufficient interactions with binding pockets within the catalytic site to achieve potency with small molecules <500 Da while also maintaining oral drug like properties, including solubility, stability, and permeability.

Under physiological conditions, trypsin like protease zymogens exist in equilibrium between an accessible active site conformation (E form) and an active site conformation that is not fully accessible to substrate (E* form) (Gohara and Cera, 2011). A proposed homology model of the S1 substrate specificity pocket of rFXII-T based on the crystal structure of a closely related enzyme, tissue plasminogen activator (tPA), suggests that Gln156 in the zymogen stabilizes the open accessible active site E form conformation by forming hydrogen bonds with Asp194 (Shamanaev et al., 2021) and allows the active conformation of the catalytic site. The X-ray crystal structure of human tPA suggests that Lys156 promotes the intrinsic activity of single-chain tPA (Renatus et al., 1997). For FXII, the Gln156 does not seem to be as effective in stabilizing an open active site as Lys156 in tPA, and the catalytic efficiency of rFXII-T for prekallikrein activation is 50,000-fold less than that of rFXIIa (Shamanaev et al., 2021). The E form is stabilized with structural changes upon cleavage at activation sites or interaction with polyanionic polymers like PolyP, which facilitates prekallikrein cleavage (Shamanaev et al., 2022).

The comparable Km of H-D-Pro-Phe-Arg-AFC for rFXIIa and rFXII-T suggests that their active sites are structurally similar, or that substrate binding forces the active sites into similar active conformations. The observation of similar IC_50_s of KV998086 for 1 nM recombinant rFXIIa (IC_50_ 21 nM) and 200 nM rFXII-T (IC_50_ 31 nM) may provide insight on the accessible conformation in the later. The comparible IC_50_s across a panel of inhibitors for rFXIIa and rFXII-T indicate that the catalytic domain structure contributing to inhibitor binding in FXIIa also exists in the accessible E form of rFXII-T. Moreover, if the affinity of KV998086 for FXIIa and the E form of rFXII-T are similar then these data would suggest that approximately 1 nM (0.5%) of the 200 nM rFXII-T in solution is in the accessible conformation for both inhibitor and substrate. The catalytic efficiency K_cat_/K_m_ was 0.431 nM^−1^ min^−1^ for rFXIIa and 7.19 × 10^−5^ nM^−1^ min^−1^ for rFXII-T in the amidolytic enzyme assays, indicating that the protease activity of rFXII-T is several orders of magnitude less (∼6,000 fold) than that of rFXIIa. Previously, a 50,000-fold difference has been reported for the protease activities of rFXIIa vs. rFXII-T, with their respective Kcat/Km as 0.26 nM^−1^ min^−1^ and 0.46 × 10^−5^ nM^−1^ min^−1^ (Shamanaev et al., 2021). Based on an estimation that 0.5% of the rFXII-T is in the E form (active accessible site), the E form catalytic efficiency would be ∼0.016 ± 0.004 nM^−1^ min^−1^. Hence, the catalytic efficiency of rFXIIa is 30 ± 8.5 fold greater than that of rFXII-T in the E form for fluorescent substrate H-D-Pro-Phe-Arg-AFC. Addition of PolyP to rFXII-T increases its cleavage of prekallikrein (Figure 4A), which is consistent with a template mechanism in which enzyme and substrate bind in proximity to each other through their respective non catalytic heavy chains (Shamanaev et al., 2022).

**FIGURE 7:**
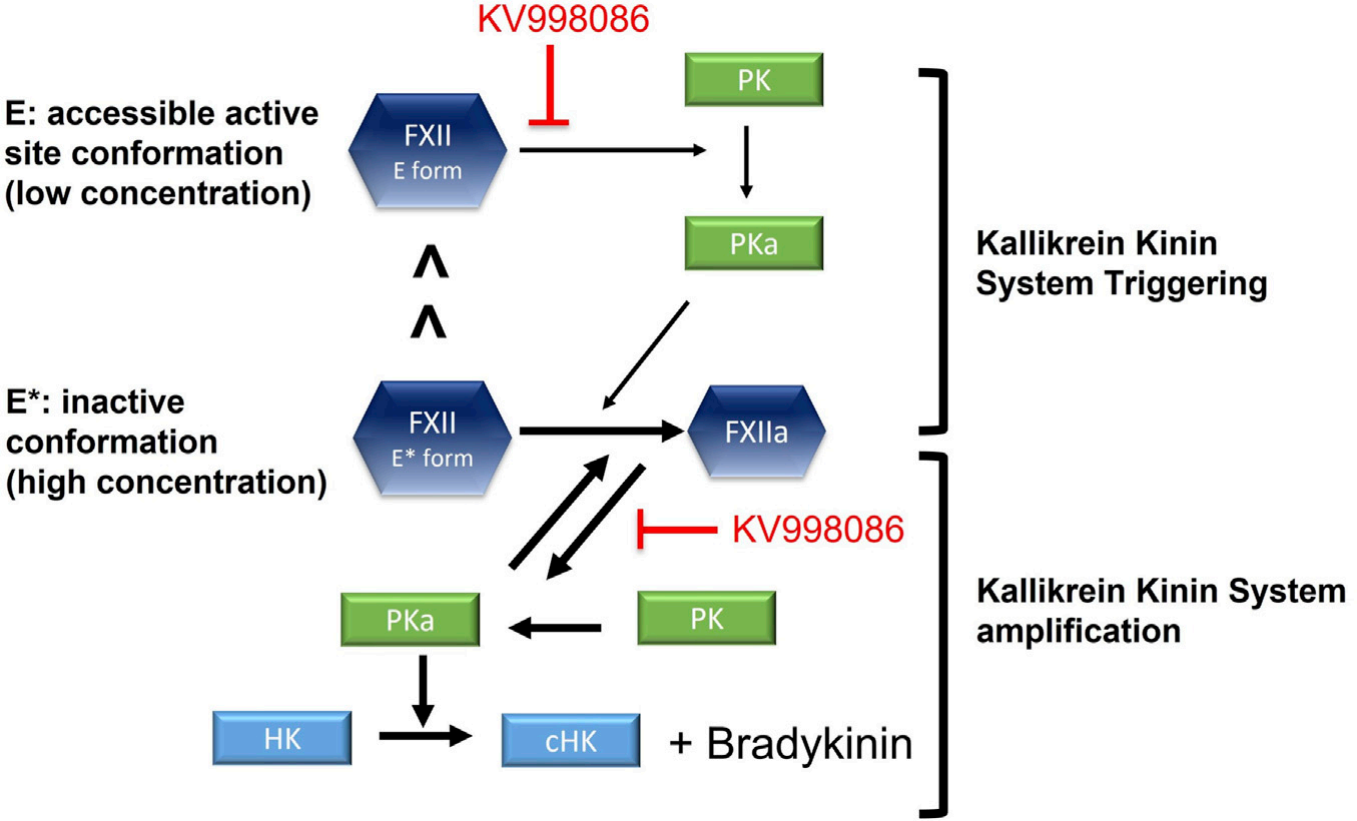
Schematic shows the effects of FXIIa inhibitor KV998086 on single chain FXII zymogen and FXIIa mediated initiation and amplification of KKS activation. In solution, a small proportion of single chain FXII is in its active conformation (E form) compared with its inactive conformation (E* form).

The low enzyme activity of the FXII E form together with its relatively low abundance may contribute to basal KKS activation and bradykinin generation under physiological conditions. Interactions of FXII with an activating surface in a tissue presenting an HAE attack trigger will facilitate its activation by PKa, and increase the PK activation by FXIIa to inititate and sustain the positive feedback process that mediates KKS amplification (Ivanov et al., 2017). Thus small molecule FXIIa inhibitors, including KV998086, could inhibit both the initation and amplification of KKS activity (Figure 7). A crystal structure for single-chain FXII zymogen is currently not available. The presence of both accessable (E) and inaccessible (E*) conformations of the active site of FXII could complicate structural studies of it accessible conformation, especially if the relative proportion of the protein in this conformation is small. Zymogen single-chain prekallikrein has also been reported to exhibit intrinsic low enzymatic activity and promotes reciprocal activation of FXII (Ivanov et al., 2020). The relative contributions of the zymogenic enzymatic activities of FXII and prekallikrein to initiating KKS activation *in vivo* are not yet available.

KV998086 is highly absorbed, with oral bioavailability >75% in multiple species with a prolonged exposure profile in plasma consistent with its relatively long oral half-life. This compound potently inhibits both human and mouse FXIIa enzyme activity (IC_50_ < 10 nM) and its pharmacology was characterized in established human plasma-based assays for the KKS and in preclinical models of angioedema. We show that KV998086 inhibits HK cleavage and the generation of PKa and FXIIa in DXS-stimulated human plasma. The protective effects of KV998086 on blocking KKS activation were comparable with the effects of the PKa inhibitor sebetralstat in these human plasma-based assays (Duckworth et al., 2022). Injection of CG into the hind paw of mice resulted in activation of KKS with near-complete consumption of circulating HK, which provided an opportunity to measure KV998086 target engagement with the KKS *in vivo*. In studies using Alzet osmotic pumps, we have shown that a plasma concentration of 165 ng/mL KV998086 was as effective as complete FXII deficiency in protection against CG-induced KKS activation and edema, and captopril-stimulated vascular permeability.

In summary, small molecule FXIIa inhibitors designed to target its active site also inhibit the enzymatic activity of FXII zymogen. We have identified a novel, potent, selective, and oral small molecule FXIIa/FXII inhibitor, KV998086, that provides protection against KKS activation and edema in preclinical models. These findings suggest that oral FXIIa inhibitors, directed against its active site, may provide a non-invasive therapeutic approach to suppress FXII zymogen mediated triggering of the KKS, as well as its amplification by FXIIa, which could contribute to HAE attacks and other FXIIa-mediated diseases.

## Data Availability

The original contributions presented in the study are included in the article/Supplementary Material, further inquiries can be directed to the corresponding author.

## Supplementary material

SUPPLEMENTARY TABLE S1 KV998086 protease selectivity. IC_50_ (half maximal inhibitory concentration) values for KV998086 inhibition of Factor XIIa and related proteases in isolated enzyme kinetic fluorogenic and chromogenic substrate assays.

SUPPLEMENTARY TABLE S2 KV998086 bioavailability. Fraction of KV998086 unbound in plasma (fup). Half-life and bioavailability of KV998086 in non-clinical species.

SUPPLEMENTARY FIGURE S1 Representative graph showing dose effect of KV998086 on mouse pFXIIa amidolytic activity measured using a fluorogenic substrate. Data are plotted as percent of control related to FXIIa activity in the absence of KV998086.

SUPPLEMENTARY FIGURE S2 Pharmacokinetic profiles following a single oral gavage of KV998086 in male C57BL/6J mice **(A)**, male Beagle dogs **(B)**, male non-human (cynomolgus) primates (NHP) **(C)** and male Sprague Dawley rats **(D)**.

SUPPLEMENTARY FIGURE S3 Effects of KV998086 on carrageenan (CG) stimulated paw thickness. **(A)** Effect of KV998086 administered via osmotic pumps upon paw thickness post 2% CG injection at 24 hours. **(B)** Effect of oral KV998086 at 45mg/kg in WT mice upon paw thickness post 0.5% CG injection at 2 hours compared to responses in FXII-/- mice.

SUPPLEMENTARY FIGURE S4 Plasma concentrations of KV998086 administered by subcutaneously implanted osmotic pump in mice. KV998086 was delivered at 2.4, 7.2 and 18 mg/kg/day using Alzet osmotic pump 1003D. Blood samples were collected at 48 hours and plasma concentration of KV998086 was measured by LC-MS/MS.
