# Supplementary figures and images for: Oral FXIIa inhibitor KV998086 suppresses FXIIa and single chain FXII mediated kallikrein kinin system activation
Source: Front Pharmacol. 2023 Dec 19;14:1287487. doi: 10.3389/fphar.2023.1287487 (PMC10766353; doi:10.3389/fphar.2023.1287487)

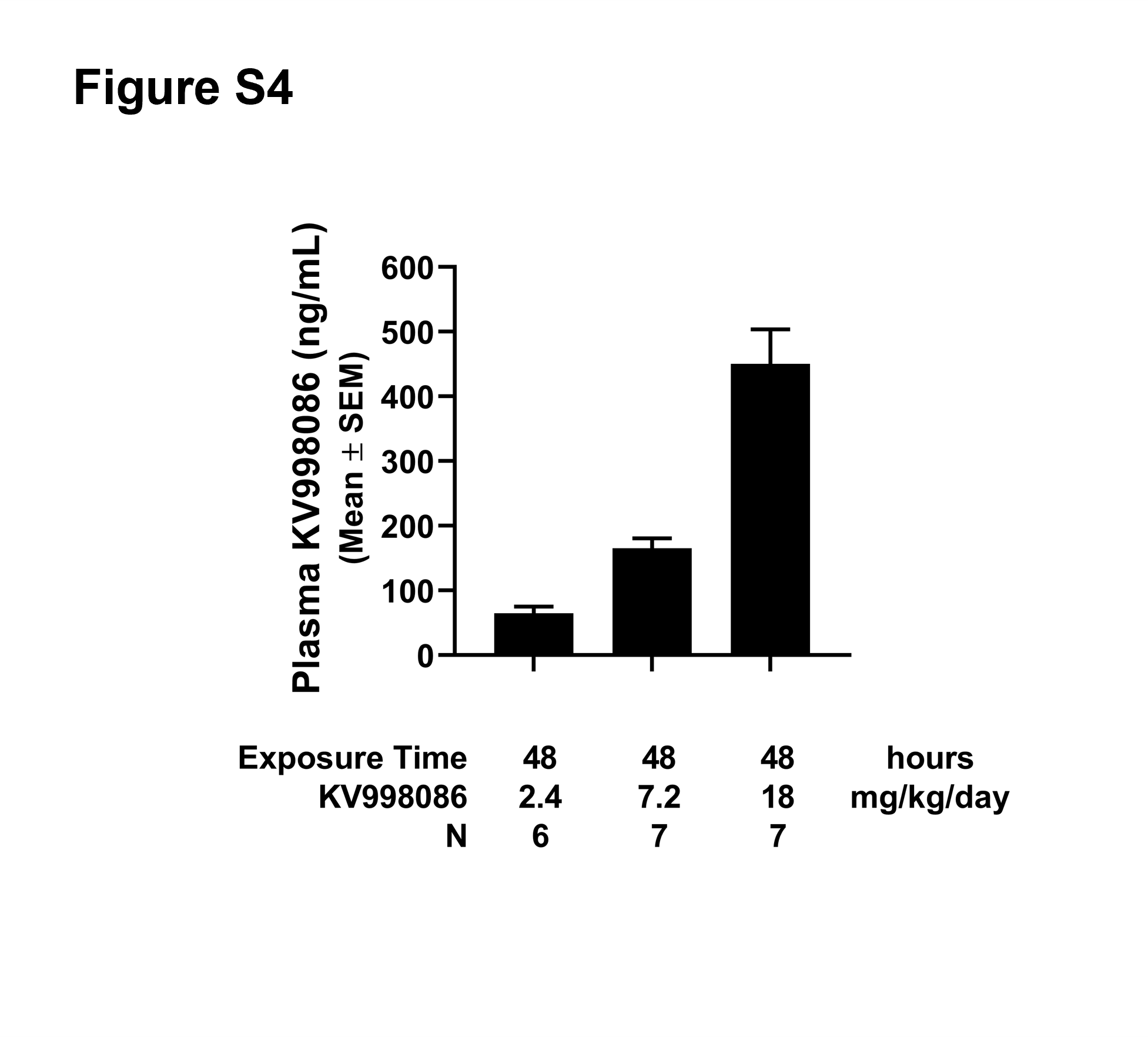

Supplement: Supplementary file 1 [file Image6.TIF]

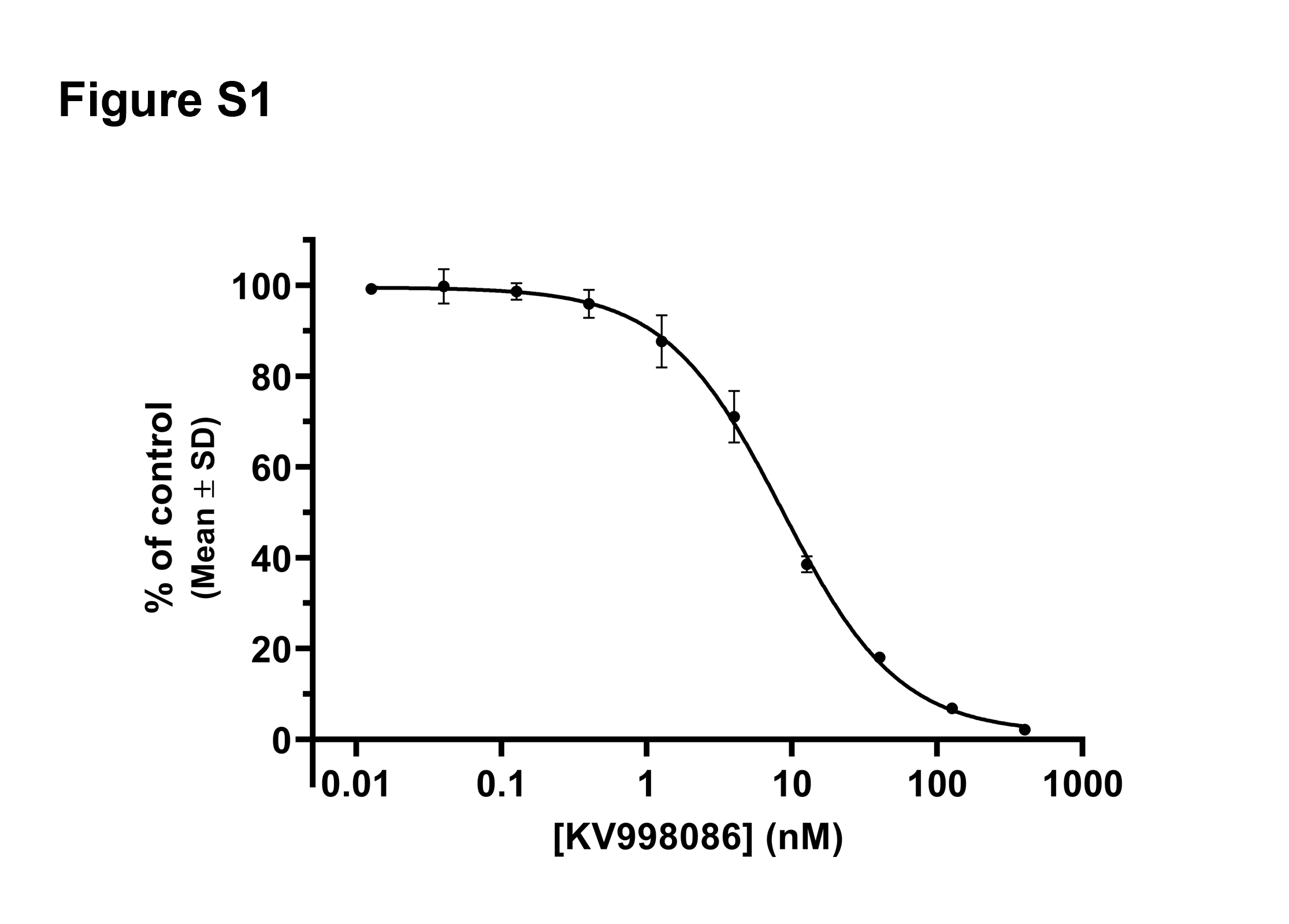

Supplement: Supplementary file 2 [file Image3.TIF]

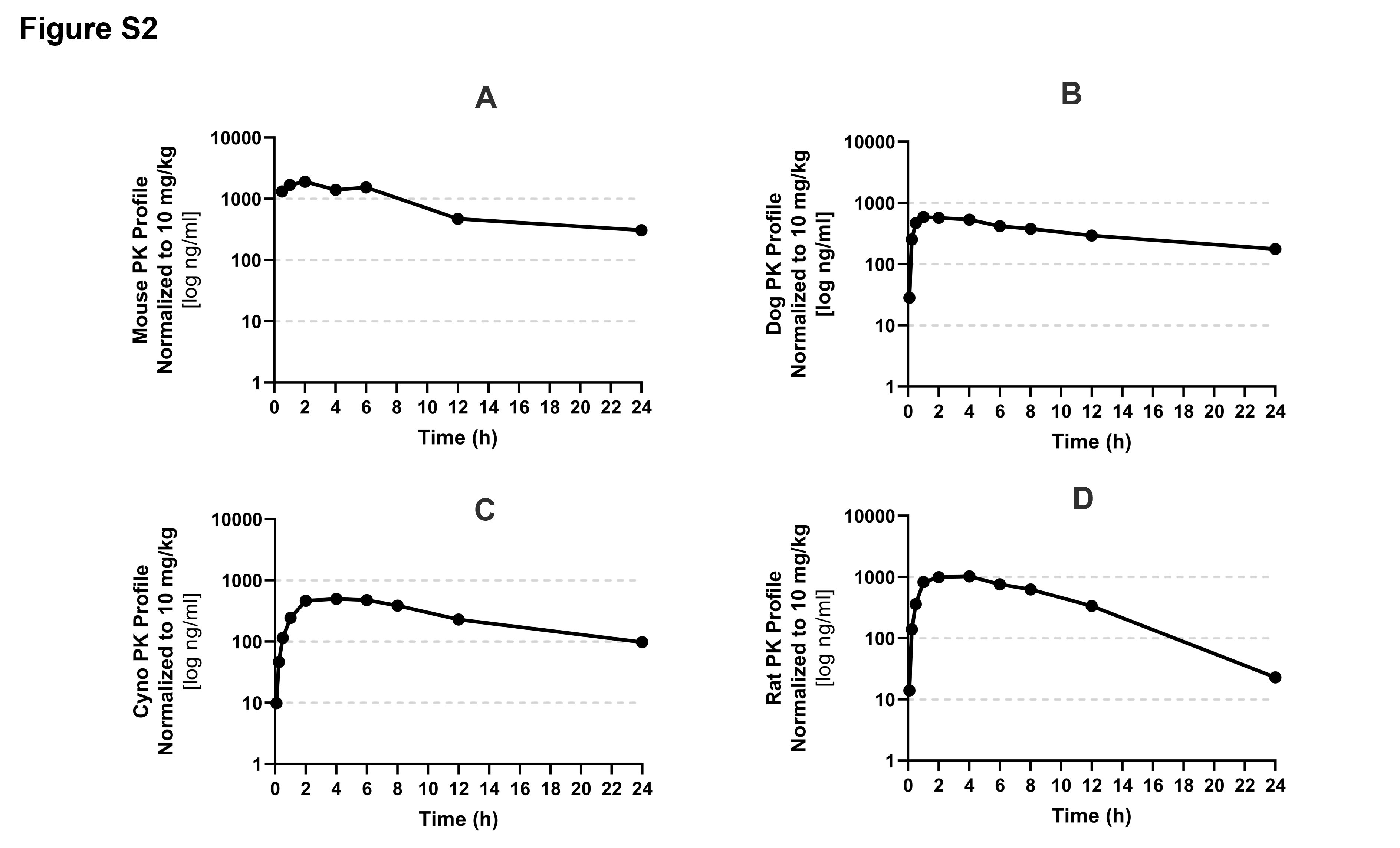

Supplement: Supplementary file 3 [file Image4.TIF]

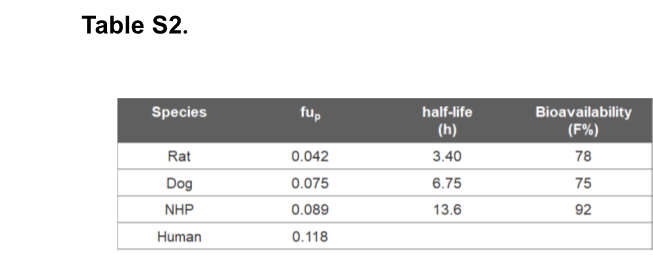

Supplement: Supplementary file 4 [file Image2.TIF]

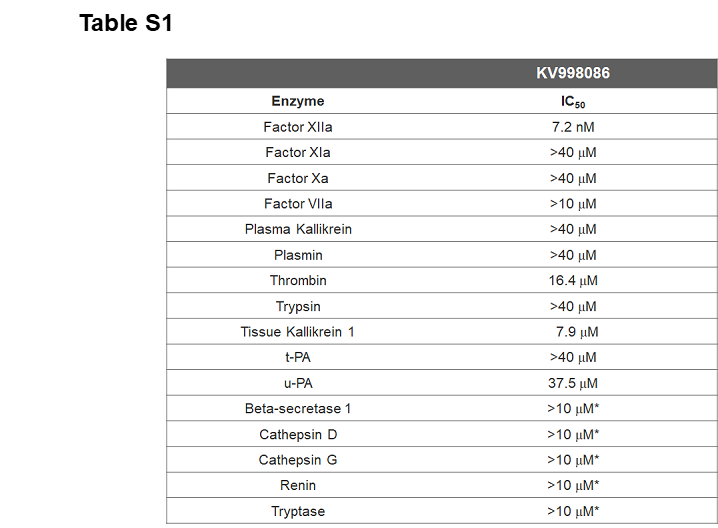

Supplement: Supplementary file 5 [file Image1.TIF]

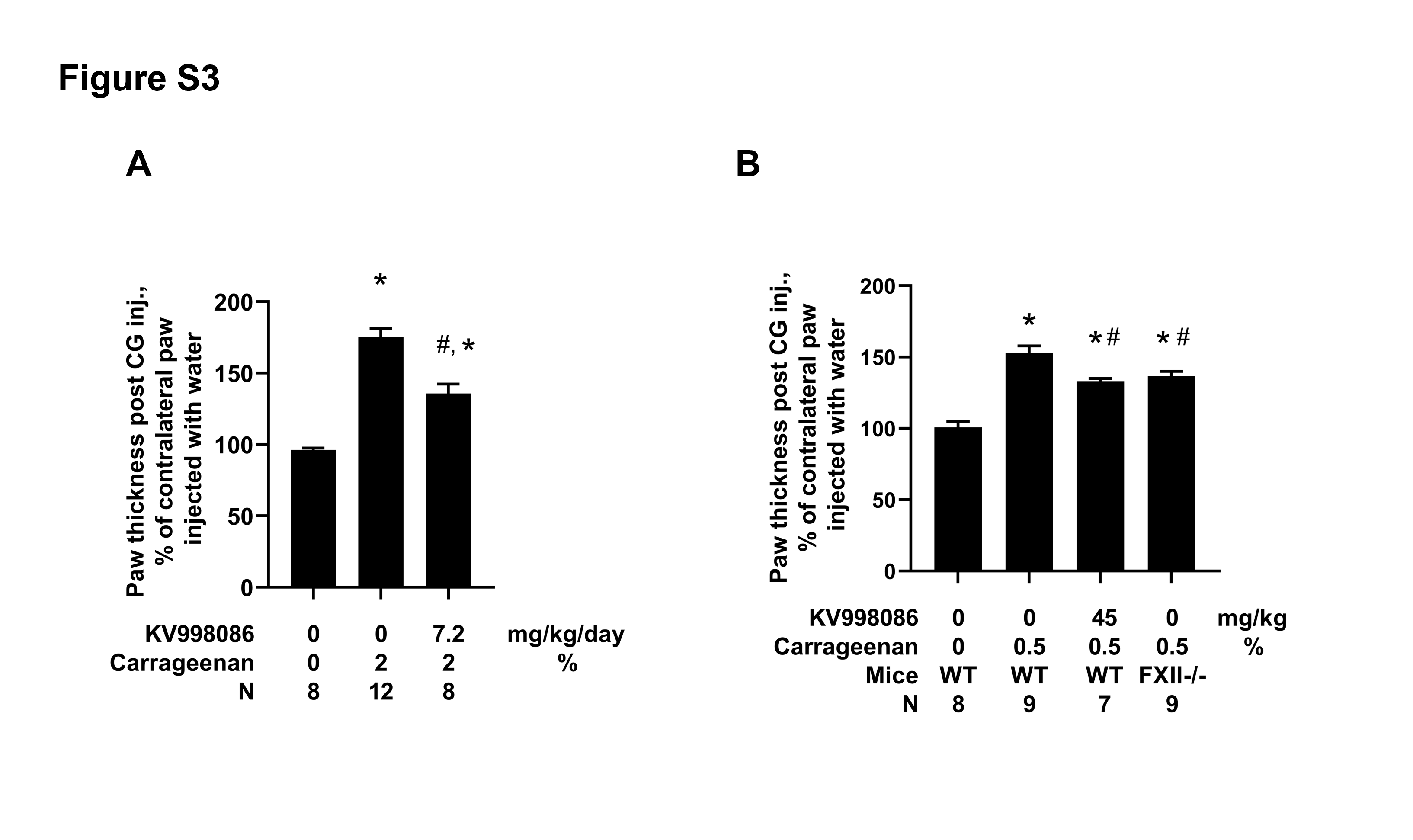

Supplement: Supplementary file 6 [file Image5.TIF]
