# Supplementary material for: Oral FXIIa inhibitor KV998086 suppresses FXIIa and single chain FXII mediated kallikrein kinin system activation
Source: Front Pharmacol. 2023 Dec 19;14:1287487. doi: 10.3389/fphar.2023.1287487 (PMC10766353; doi:10.3389/fphar.2023.1287487)
Supplement: Supplementary file 7 [file DataSheet1.DOCX]

**SUPPLEMENTAL METHODS**

**KV998086 Selectivity**

Isolated human serine proteases factor XIa, factor Xa, PKa, tissue kallikrein 1 (*KLK1*), plasmin, thrombin, trypsin, tissue plasminogen activator (t-PA) and urokinase plasminogen activator (u-PA), and corresponding fluorogenic substrates were commercially sourced (FXa, FXIa (Enzyme Research Laboratories, UK), tissue kallikrein 1, t-PA and u-PA (R&D Systems, UK), Plasmin (Fisher Scientific, UK), Thrombin (Roche, UK) and Trypsin (Sigma Aldrich, UK). Protease activity and KV998086 IC_50_ were determined using methodology described for FXIIa, at concentrations up to 40 µM, in n=3 experiments. Eurofins Discovery (Taiwan) performed assays on isolated human serine proteases factor VIIa, beta-secretase 1, cathepsin D, cathepsin G, renin, and tryptase. Inhibition was determined at a single concentration of 10 µM.

**Plasma protein binding (PPB)**

Plasma free fraction was determined using rapid equilibrium dialysis system (Thermo Scientific, UK). KV998086 was tested at 1 µM in rat, dog, non-human (cynomolgus) primates (NHP) and human plasma (BioIVT, UK) and dialysed against a phosphate buffer for 5 hours at 37 ˚C. Quantitation of KV998086 was performed via liquid chromatography tandem mass spectrometry (LC-MS/MS).

**Pharmacokinetic studies**

Pharmacokinetic (PK) studies were performed to assess plasma concentrations of KV998086 following a single intravenous (IV) or oral (po) dose in male Sprague Dawley rats (7 – 8 weeks of age and 250 – 350 g weight), male Beagle dogs (0.8 – 5 years of age and 8 – 15 kg weight) and male non-human (cynomolgus) primates (NHP) (40 months of age and 4.45 – 5.18 kg weight). Pharmaron UK, Ltd performed PK studies in the rat and dog. Labcorp UK, Ltd performed PK studies in NHP. All scientific procedures on these animals were conducted in the United Kingdom in accordance with the Animals (Scientific Procedures) Act 1986. Four naïve rats were given either a single nominal dose: 1 mg/kg IV (n=2) or 5 mg/kg po (n=2) of KV998086 formulated in 10% DMSO, 10% Cremophor EL and 80% water. Two non- naïve dogs and two non- naïve NHP were given a single nominal dose: 0.5 mg/kg IV and following a wash-out period of at least 7 days, a single nominal dose of 5 mg/kg po of KV998086 formulated in 10% DMSO (aq).

For the rat PK,. blood samples were obtained under anesthesia from a lateral tail vein at 2, 5, 15 and 30 minutes then 1, 2, 4, 6, 8, 12 and 24 hours following either IV administration or oral administration into sodium heparin coated containers. For dogs and NHP, blood samples were taken from the jugular vein, or femoral or saphenous vein, respectively, at 2, 5, 15 and 30 minutes and 1, 2, 4, 6 and 12 hours following IV administration or oral administration into 3.2% sodium citrate containers. Following collection, blood samples were centrifuged at 4 ˚C at 10000 g for 2 minutes for the rat and dog, and at 4 ^o^C at 1500 g for 10 minutes for the NHP and plasma was collected. Quantitation of KV998086 was performed against an extracted calibration line via LC-MS/MS. A non-compartmental analysis using the quantitative plasma concentration data was performed using Phoenix WinNonlin v8.3.

Mouse PK studies were performed by KalVista Pharmaceuticals. A single nominal oral dose of 45 mg/kg of KV998086 formulated at 6 mg/mL in 10% DMSO, 10% Cremophor EL and 80% water was delivered by gavage to male C57BL/6J mice. Blood was collected from the descending vena cava via 26G 1 cc syringe containing 3.2% sodium citrate at 30 minutes and 1, 2, 4, 6, 12 and 24 hours following oral administration. The blood was centrifuged at 3000rpm at 21˚C for 12 minutes and plasma was collected. The plasma was stored at -80˚C until analysis to quantify concentration of KV998086 by LC-MS/MS.
